# Descriptive review of current practices and prognostic factors in patients with ovarian cancer treated by pressurized intraperitoneal aerosol chemotherapy (PIPAC): a multicentric, retrospective, cohort of 234 patients

**DOI:** 10.3389/fonc.2023.1204886

**Published:** 2023-08-24

**Authors:** Amaniel Kefleyesus, Aditi Bhatt, Cecilia Escayola, Vladimir Khomyakov, Martin Hübner, Marc A. Reymond, René Thieme, Olivia Sgarbura, Wouter Willaert, Wim Ceelen, Andrea Di Giorgio, Giuseppe Vizzielli, Olivier Glehen, Manuela Robella, Naoual Bakrin

**Affiliations:** ^1^ Department of Surgical Oncology, Lyon University Hospital, Centre Hospitalier Lyon-Sud, Lyon, France; ^2^ Department of Visceral Surgery, Lausanne University Hospital CHUV, University of Lausanne (UNIL), Lausanne, Switzerland; ^3^ Department of Surgical Oncology, Zydus Hospital, Ahmedabad, India; ^4^ Division of Gynaecologic Surgery, Clinica del Pilar, Barcelona, Spain; ^5^ Moscow Research Oncological Institute named after (n. a.) Pyotr Alexanderovich (P. A.) Herzen, Thoracoabdominal, Moscow, Russia; ^6^ Department of General and Transplant Surgery , University Hospital Tübingen, Tübingen, Germany; ^7^ Department of Visceral, Transplant, Thoracic and Vascular Surgery, University Hospital of Leipzig, Leipzig, Germany; ^8^ Department of Surgical Oncology, Cancer Institute Montpellier (ICM), University of Montpellier, Montpellier, France; ^9^ Department of GI Surgery, Ghent University Hospital, Ghent, Belgium; ^10^ Cancer Research Institute Ghent (CRIG), Ghent University Hospital, Ghent, Belgium; ^11^ Surgical Unit of Peritoneum and Retroperitoneum, Fondazione Policlinico, Universitario A. Gemelli Scientific Institute for Research, Hospitalization and Healthcare (IRCCS), Rome, Italy; ^12^ Department of Obstetrics and Gynecology, Fondazione Policlinico, Universitario A. Gemelli Scientific Institute for Research, Hospitalization and Healthcare (IRCCS), Rome, Italy; ^13^ Unit of Surgical Oncology, Candiolo Cancer Institute, Fondazione Policlinico (FPO)-Scientific Institute for Research, Hospitalization and Healthcare (IRCCS), Candiolo, Italy

**Keywords:** peritoneal metastases, ovarian cancer, PIPAC, prognostic factors, platinum sensitivity

## Abstract

**Introduction:**

Ovarian cancer (OC) is the primary cause of mortality in women diagnosed with gynecological cancer. Our study assessed pressurized intraperitoneal aerosol chemotherapy (PIPAC) as treatment for peritoneal surface metastases (PSM) from recurrent or progressive OC and conducted survival analyses to identify prognostic factors.

**Material and methods:**

This retrospective cohort study, conducted across 18 international centers, analyzed the clinical practices of patients receiving palliative treatment for PSM from OC who underwent PIPAC. All patients were initially treated appropriately outside any clinical trial setting. Feasibility, safety, and morbidity were evaluated along with objective endpoints of oncological response. Multivariate analysis identified prognostic factors for OS and PFS.

**Results:**

From 2015-2020, 234 consecutive patients were studied, from which 192 patients were included and stratified by platinum sensitivity for analysis. Patients with early recurrence, within one postoperative month, were excluded. Baseline characteristics were similar between the groups regarding platinum sensitivity (platinum sensitive (PS) and resistant (PR)), but chemotherapy frequency differed, as did PCI before PIPAC. Median PCI decreased in both groups after three cycles of PIPAC (PS 16 vs. 12, p *<* 0.001; PR 24 vs. 20, p = 0.009). Overall morbidity was 22%, with few severe complications (4-8%) or mortality (0-3%). Higher pathological response and longer OS (22 vs. 11m, p = 0.012) and PFS (12 vs. 7m, p = 0.033) were observed in the PS group. Multivariate analysis (OS/PFS) identified ascites (HR 4.02, p *<* 0.001/5.22, p *<* 0.001), positive cytology at first PIPAC (HR 3.91, p = 0.002/1.96, p = 0.035), and *≥* 3 PIPACs (HR 0.30, p = 0.002/0.48, p = 0.017) as independent prognostic factors of overall survival/progression-free survival.

**Conclusions:**

With low morbidity and mortality rates, PIPAC is a safe option for palliative treatment of advanced ovarian cancer. Promising results were observed after 3 PIPAC, which did improve the peritoneal burden. However, further research is needed to evaluate the potential role of PIPAC as an independent prognostic factor.

## 1 Introduction

Epithelial ovarian cancer (EOC) is the most lethal gynecological cancer, affecting more than 300,000 new cases annually worldwide. Despite its rare incidence, it is burdened with a high mortality rate of more than 200,000 deaths in 2020 (1, 2). Despite a high initial response rate after first-line chemotherapy, only 40-60% result in a complete response (3). The 60-70% of diagnoses occur at the stage of peritoneal carcinosis and the natural course includes sequential relapses, which leads to an ever-increasing probability of platinum resistance relapse (4–7). In addition, several studies have shown the feasibility, safety, and good tolerance of PIPAC (8–10). In the palliative setting after first-line chemotherapy, pressurized intraperitoneal aerosol chemotherapy (PIPAC) with a cisplatin-doxorubicin protocol is currently a safe option. The oncological efficacy has yet to be evaluated [Bakrin et al. (11); Tempfer et al. (12)]. The present study aimed to provide a descriptive report of the current practices in the management of PSM in recurrent or first-line progressive EOC treated with PIPAC in a palliative setting. This study aimed to outline prognostic factors for survival and progression.

## 2 Materials and methods

### 2.1 Patient’s selection

This multicenter international retrospective analysis from 18 centers included 234 patients diagnosed with PSM from EOC, irrespective of the histologic subtype, between July 2015 and March 2020. Eligibility criteria were as follows: adult patients having palliative treatment with PIPAC, recurrent EOC, tumor board approval for PIPAC, and signed surgical informed consent. Patients with extraperitoneal metastases were excluded from this study. Recurrence was defined according to the timing of recurrence. Patients were described as “platinum-sensitive” (PS) if recurrence occurred more than 6 months after the completion of the initial treatment. Early recurrence before 6 months was considered “platinum-resistant” (PR) (13).

### 2.2 Morphological and pathological responses evaluation

Treatment strategies were defined and regularly reassessed during multidisciplinary team (MDT) meetings. Following 3 or at least 2 PIPAC, the morphological and pathological responses were confirmed during the MDT meeting, based on expert radiologists’ and pathologists’ reviews. Morphological response was described according standard and objective radiological response criteria described using the Response Evaluation Criteria In Solid Tumors (RECIST) version 1.1 (14). The types of response described were: complete response, partial response, progressive disease, and stable disease. Pathological response was described according the peritoneal regression grading score (PRGS); no residual cancer cells in all specimens (PRGS 1: complete response), 1 to 49% residual cancer cells (PRGS 2: major response), *≥* 50% (PRGS 3: minor response) and finally no response (PRGS 4) (15).

### 2.3 Surgery

Eligibility for PIPAC was confirmed after a systematic exploratory laparoscopy done with a peritoneal cancer index (PCI); a sample of ascitis or peritoneal washing for cytology; peritoneal biopsies for histopathological examination; and the sufficient work space for aerosolization of the intraperitoneal chemotherapy. All PIPAC procedures were performed by expert surgeons dedicated to peritoneal metastases management. Every surgeon was specifically trained in PIPAC procedures following published standard practice and safety protocols (8, 16–18). Drugs administered during early experience were cisplatin at 7.5 mg/m2 dosage and doxorubicin at 1.5 mg/m2. Furtherly those dosages were upgraded to respectively 10.5 and 2.1 mg/m2 with supporting safety and encouraging data (12) Postoperative morbidity and mortality were recorded according to Dindo-Clavien classification (19).

### 2.4 Statistical analysis

Student’s t-test was used for continuous variables, as a parametric test, and the McNemar test for categorical variables, as a non-parametric test. Fisher’s exact test was used for comparisons between the groups. The Mann-Whitney U test was used as a non-parametric test for comparisons between independent variables without a Gaussian distribution and the equality of variance assumption. Univariate and multivariate survival analyses were conducted using Cox model regression. Missing data was handled without imputation. Survival endpoints were defined as the time between the PSM diagnosis date and first PIPAC until death from any cause for overall survival (OS), and disease progression (PFS) expressed by radiological recurrence, symptomatic disease progression or death. The potential impact of PIPAC on OS is further supported by the fact that our study focused on patients with previously controlled disease through systemic chemotherapy, without the presence of extraperitoneal disease. By selecting patients with controlled disease, we aimed to evaluate the additional benefits of PIPAC in a specific subset where the peritoneal cavity remained a significant site of disease burden. The assumption underlying our study is that by targeting and controlling the spread of cancer within the peritoneal cavity, PIPAC may further contribute to improved survival outcomes in this particular context. The hazard ratios (HR) for PIPAC, clinical symptoms, and PCI before PIPAC, and the confounding factors were estimated with 95% confidence interval (95% CI) through the Cox regression multivariate model. The assumption of hazard proportionality over time was confirmed in the selected model. The best regression model was chosen with the literature based known prognostic factors, with the “stats” R package. Survival rates were estimated using the Kaplan-Meier method and compared using the log-rank test. Analysis was performed using RStudio Software (RStudio: Integrated Development for R. PBC, Boston, MA, 2020). Statistical significance was set at a two-sided p-value of *<* 0.05.

### 2.5 Compliance with ethical standards

This study was conducted in compliance with international standards for research practice and reporting. Written informed consent was obtained from all included patients. All data were de-identified and anonymized prior to analysis. A retrospective analysis was approved by the local institutional review board of each participating center and was conducted in compliance with the STROBE criteria (www.strobe-statement.org).

## 3 Results

### 3.1 Baseline characteristics

A total of 234 consecutive patients were treated with palliative intent for OC. Patients without sufficient data were excluded (n = 20, 9%). After excluding early recurrence, 192 patients (82%) had recurrence after receiving initial treatment, including chemotherapy ± surgery, and 22 patients (9%) were treated frontline after initial chemotherapy and unrespectability. A flow chart of the included patients is shown in **Figure S1** (**Supplementary Materials**). Baseline patient characteristics are shown in a comparative cross-table by platinum sensitivity group, with the majority of patients in the platinum-sensitive group (116 patients, 60%). Patients were comparable in terms of comorbidities, performance status, and delay of management between PSM diagnosis and 1st PIPAC cycle. The patients differed in age and primary tumor subtype. Patients in the PS group were older (median 64 vs. 60 years, p = 0.024) and more heterogeneous regarding histologic subtypes compared to the PR group. Further details are provided in **Table 1**.

**Table 1:** Baseline characteristics.

Characteristic	N	Platinum sensitive N = 116 (60%)^1^	Platinum resistant N = 76 (40%)^1^	p-value^2^
Age	192	64 (57, 70)	60 (53, 67)	0.024
BMI	162	23.5 (20.7, 26.8)	23.8 (20.6, 27.3)	0.39
Missing		20	10	
ASA	179			0.34
1		15 (14%)	9 (13%)	
2		61 (55%)	33 (49%)	
3		35 (32%)	24 (35%)	
4		0 (0%)	2 (2.9%)	
Missing		5	8	
ECOG	172			0.65
0		57 (56%)	34 (48%)	
1		32 (32%)	26 (37%)	
2		10 (9.9%)	9 (13%)	
3		1 (1.0%)	2 (2.8%)	
4		1 (1.0%)	0 (0%)	
Missing		15	5	
Primary tumor subtype	174			0.038
Serous adenocarcinoma		91 (92%)	71 (95%)	
Mucinous		6 (6.1%)	0 (0%)	
Other		2 (2.0%)	4 (5.3%)	
Missing		17	1	
Delay between PSM-PIPAC*	178	22 (9, 40)	16 (8, 32)	0.23
Missing		12	2	

^1^ Median (IQR); n (%).

^2^ Wilcoxon rank sum test; Fisher's exact test.

*Delay since peritoneal metastases (PSM) diagnostic and 1st PIPAC.

ASA, American Society of Anesthesiology classification; ECOG, European Eastern Cooperative Oncology Group for performance status scale; BMI, body mass index (kg/m^2^).

### 3.2 Past chemotherapy history

Regarding the number of previous chemotherapy lines or maintenance treatments (bevacizumab) received, PR group (n=28/48, 58%) had more bidirectional treatment in combination with PIPAC cycles compared to PS group (n=27/79, 34%, p = 0.008). About 2/3 and 1/3 of patients had undergone prior PIPAC initiation respectively 3 and 2 lines of chemotherapy. Further details are presented in **Table 2**.

**Table 2:** Past chemotherapy history.

Characteristic	N	Platinum sensitive N = 116 (60%)^1^	Platinum resistant N = 76 (40%)^1^	p-value^2^
1^st^ line	190	115	75	>0.99
Missing		1	1	
1^st^ line (type)	186			0.30
Platinum based		100 (88%)	61 (84%)	
Bevacizumab + CT		11 (9.7%)	12 (16%)	
Other		2 (1.8%)	0 (0%)	
Missing		3	3	
2^nd^ line	187	94 (82%)	65 (89%)	0.22
Missing		2	3	
2^nd^ line (type)	182			0.009
Platinum based		43 (38%)	17 (24%)	
Bevacizumab + CT		37 (33%)	27 (39%)	
Other		12 (11%)	19 (27%)	
No CT		20 (18%)	7 (10%)	
Missing		4	6	
3^rd^ line	180	60 (55%)	42 (60%)	0.47
Missing		6	6	
3^rd^ line (type)	178			0.23
Platinum based		16 (15%)	9 (13%)	
Bevacizumab + CT		11 (10%)	6 (8.6%)	
Other		26 (24%)	27 (39%)	
No CT		55 (51%)	28 (40%)	
Missing		8	6	
Systemic chemotherapy (cycles)	123	14 (8, 20)	13 (10, 18)	0.70
Missing		46	23	
PARPi (before PIPAC)	159	10 (11%)	5 (7.8%)	0.57
Missing		21	12	
Bidirectional chemotherapy (IV-IP)	127	27 (34%)	28 (58%)	0.008
Missing		37	28	

^1^ n (%); Median (IQR).

^2^ Fisher's exact test; Pearson's Chi-squared test; Wilcoxon rank sum test.

CT, platinum based or other chemotherapy; PARPi, poly ADP ribose polymerase inhibitor.

### 3.3 Surgical data

Past surgical history analysis showed differences among groups, with a history of CRS greater in the PR group (80% vs. 67%, p = 0.047); however, the PS group showed more cases with a history of HIPEC (12% vs. 1.3%, p = 0.007). The peritoneal burden in the PR group was higher during the PCI evaluation at 1st PIPAC, with a higher median PCI value (16 vs. 24, p *<* 0.001). The PS group had significantly more PIPAC cycles (median, 3 vs. 2 cycles; p = 0.016). Follow-up after the 3rd PIPAC showed a significant decrease in initial PCI in both groups, with a median of 16 vs. 24 at 1st PIPAC (p *<* 0.001), and 12 vs. 20 after 3rd PIPAC (p = 0.009), respectively, for the PS vs. PR groups. The results detailed in **Table 3** also showed overall surgical morbidity of 19 vs. 28% (p = 0.16), with low open laparoscopy-related morbidity (0.92% vs. 2.7%, p = 0.56), low severe postoperative complications (4.3 vs. 7.9%, p = 0.35), and in-hospital mortality (2.6 vs. 0%, p = 0.28), respectively, for the PS and PR groups. Renal parameters were closely monitored throughout the treatment course, and no instances of renal failure related to cisplatin use were observed for this cohort.

**Table 3:** Surgical data.

Characteristic	N	Platinum sensitive N = 116 (60%)^1^	Platinum resistant N = 76 (40%)^1^	p-value^2^
History of HIPEC	191			0.007
None		102 (88%)	74 (99%)	
Yes		14 (12%)	1 (1.3%)	
Missing		0	1	
History of CRS	185			0.047
None		36 (33%)	15 (20%)	
Yes		73 (67%)	61 (80%)	
Missing		7	0	
PCI (at 1st PIPAC)	183	16 (9, 24)	24 (17, 30)	<0.001
Missing		9	0	
PCI (at 2nd or 3rd PIPAC)	128	12 (6, 19)	20 (6, 28)	0.009
Missing		36	28	
Cytology (at 1st PIPAC)	115			0.76
positive		36 (63%)	35 (60%)	
Missing		59	18	
N PIPAC (by patient)	192	3 (1, 3)	2 (1, 3)	0.016
PIPAC (2 cycles)*	192	20 (17%)	18 (24%)	0.27
PIPAC (3 cycles)*	192	66 (57%)	30 (39%)	0.018
Overall complications	192	22 (19%)	21 (28%)	0.16
Severe complications (Clavien ≥3)^#^	192	5 (4.3%)	6 (7.9%)	0.35
Open laparoscopy related^¶^	184	1 (0.9%)	2 (2.7%)	0.56
Missing		5	3	
Mortality at 30-days	191	3 (2.6%)	0 (0%)	0.28
Missing		1	0	

^1^ n (%); Median (IQR).

^2^ Pearson's Chi-squared test; Wilcoxon rank sum test; Fisher's exact test; Mann-Whitney U test.

* Patients who completed at least 2 or 3 PIPAC cycles. 1 cycle = 1 PIPAC procedure.

^#^ Clavien-Dindo classification, greater or equal than grade 3.

^¶^ Complications related to surgical access issue (e.g., small bowel perforation during open-laparoscopy).

### 3.4 Oncological response

In terms of objective assessment, the morphological evaluation at the end of PIPAC cycles showed only a tendency for more complete responses in the PS group and more stable responses in the PR group (p = 0.16) with a substantial amount of missing data (49.5%). The pathological evaluation showed a significant difference with a higher rate of complete or major response in the PS group (26% and 38% versus 8 and 32% in the PR group, respectively; p = 0.016). The details of the data are presented in **Table 4**.

**Table 4:** Oncological response.

Characteristic	N	Platinum sensitive N = 116 (60%)^1^	Platinum resistant N = 76 (40%)^1^	p-value^2^
Morphologic response (RECIST 1.1)*	100			0.16
Complete		12 (20%)	4 (9.8%)	
Partial		14 (24%)	8 (20%)	
Stable		13 (22%)	17 (41%)	
Progression		20 (34%)	12 (29%)	
Missing		57	35	
PRGS^#^	96			0.016
PRGS 1		15 (26%)	3 (7.9%)	
PRGS 2		22 (38%)	12 (32%)	
PRGS 3		19 (33%)	16 (42%)	
PRGS 4		2 (3.4%)	7 (18%)	
Missing		58	38	
Positive cytology*	64	29 (78%)	19 (70%)	0.46
Missing		79	49	

^1^ n (%).

^2^ Fisher's exact test; Pearson's Chi-squared test.

^*^ Morphological response according RECIST 1.1 criteria, after 3 PIPAC or at least 2 PIPAC.

^#^ PRGS: Pathological Regression Grading Score; 1= complete response; 2= major response (>50% fibrosis); 3=partial response (<50% fibrosis); 4=no response.

### 3.5 Follow-up

The median follow-up was 8 months (IQR 3-17) vs. 6 months (IQR 2-14) for the PS and PR groups, respectively. The overall population follow-up rate was 86.5%. The reasons for the termination of PIPAC are listed in **Table S2**. A small proportion of patients (7-8%) had to withdraw due to surgical access difficulties (multivisceral adhesions). Approximately 38% of the patients with PS and 24% of those with PR completed the planned PIPAC cycles. Between 9% and 10% of patients were eligible for CRS. Roughly 30% of patients received supportive or palliative care. The remaining 2/3 of the patients resumed systemic chemotherapy. Progression at follow-up was documented for 67% of the PS group and 81% of the PR group (see **Table S2** in **Supplementary Materials**).

### 3.6 Survival analysis

**Figure 1:**
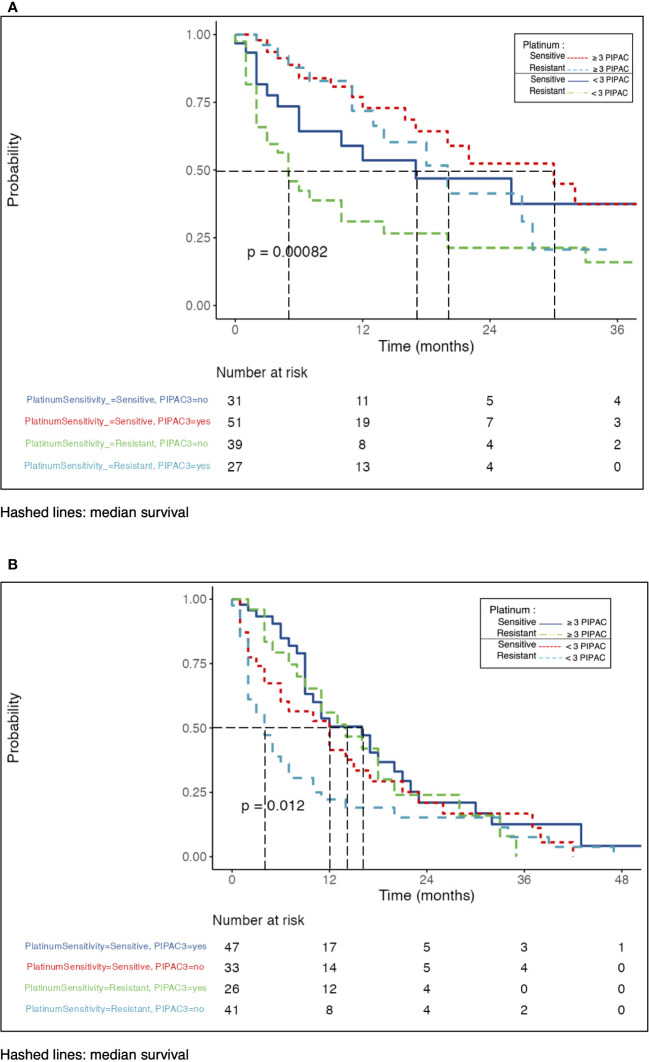
**(A)** Overall survival after PIPAC adjusted to platinum sensitivity. **(B)** Progression-free survival after PIPAC adjusted to platinum sensitivity.

Overall survival (OS) Overall survival analysis showed a median of 16 months (95%CI, 12-22). Subgroup OS analysis showed a median of 22 vs. 11 months (PS vs. PR, p = 0.012). The survival rates at 12, 24, and 36 months were 65% vs. 47%, 47% vs. 30%, and 36% vs. 19% for the PS and PR groups, respectively. OS analysis adjusted for the number of PIPACs performed revealed difference in platinum sensitivity, with a greater delta in the PR group (p = 0.002) (**Figure S2**). In the subgroup analysis, patients with three or more PIPACs showed a longer OS in the PS vs. PR group (median 30 vs. 18 months, p = 0.31). Subgroup with fewer than three PIPACs had longer OS in the PS group (median 17 vs. 5 months, p = 0.051) (**Figure 1A**).

### 3.7 Progression-free survival

The overall population PFS analysis showed a median of 10 months (95%CI, 9-13). Subgroup PFS analysis showed median of 12 vs. 7 months (PS vs. PR, p = 0.033). Survival rates at 12, 24, and 36 months were 49% vs. 35%, 22% vs. 20%, and 16% vs. 6% for the PS vs. PR groups, respectively. Comparison of PFS between groups adjusted for the number of PIPACs performed showed a significant difference in platinum sensitivity (p = 0.007) (**Figure S3**). Subgroup analysis with less than 3 PIPACs had median PFS 12 vs. 4 months (p = 0.12), in PS vs. PR-group, respectively. The subgroups with three or more PIPACs were comparable, regardless of platinum sensitivity (median 16 vs. 13 months, p = 0.47) (**Figure 1B**).

### 3.8 Multivariate survival analysis: Cox model

**Figure 2:**
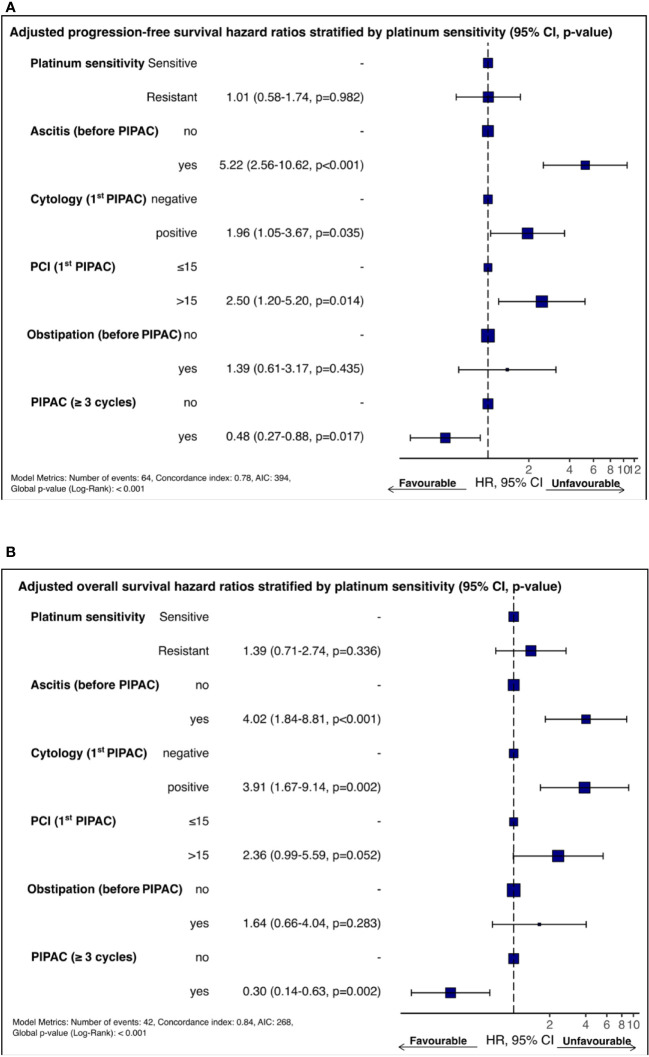
**(A)**Forest plot of adjusted OS predictors stratified by platinum sensitivity. **(B)** Forest plot of adjusted PFS predictors stratified by platinum sensitivity.

The multivariate overall survival analysis is summarized in **Figure 2A**. The OS forest plot shows the predictive factors adjusted for the key prognostic factors for survival, including platinum sensitivity. The presence of ascitis (HR = 4.02, 95% CI 1.84-8.81, p *<* 0.001) with positive cytology (HR = 3.91, 1.67-9.14, p = 0.002) at the 1st PIPAC was an independent OS prognostic factor. Performing three or more PIPACs treatments (HR = 0.3, 0.14-0.63, p = 0.002) showed to be an independent OS prognostic factor. The adjusted analysis of the predictive factors of PFS showed the same trends as OS (**Figure 2B**). The presence of ascitis (HR = 5.22, 2.56-10.62, p *<* 0.001), PCI *>* 15 (HR = 2.5, 1.2-5.2, p = 0.014), and cytology (HR = 1.960, 1.05-3.67, p = 0.035) were found to be independent unfavorable predictive factors for PFS. The completion of at least three PIPACs (HR = 0.48, 0.27-0.88, p = 0.017) was an independent factor for good prognosis regarding PFS. Detailed univariate and multivariate Cox regression analyses are depicted in **Tables S3A**, **B** (**Supplementary Material**).

## 4 Discussion

Treatment of patients with recurrent or unresectable OC remains a therapeutic challenge. An increasing number of subsequent lines of chemotherapy is associated with decreased benefits for patients. Hanker et al. showed a very diminished survival benefit of successive chemotherapy lines after the 4th recurrence (4). Moreover, the prognostic becomes poorer with PR recurrence regardless the adjunct of bevacizumab to chemotherapy as in described in the AURELIA trial, which is currently the best available treatment (PFS 6.7 months from start of 2nd line chemotherapy), or PARP inhibitor (20, 21). Intraperitoneal route for chemotherapy is a valid option largely described since Armstrong et al. work in 2006 (22, 23). PIPAC represents currently a safe and effective technique and vector of IP chemotherapy for palliative OC after failure of multiple lines of chemotherapy and targeted therapies (anti-VEGF, PARPi) (24–27).

The present study reports descriptive terms for the current practices of 12 centers around the world. The detailed analysis of postoperative morbidity and mortality found the same conclusions in the literature in terms of safety, even in patients with a history of extensive cytoreduction (8, 25). The theoretical goal of PIPAC is to stabilize intra-abdominal disease, improve QoL in case of symptoms and delay a new line of IV chemotherapy, in a palliative management setting. In our study, objective radiological and pathological evaluations were difficult to document exhaustively. This is likely due to the inconsistent availability of targets for radiological evaluations. Accessibility to specialized pathological reading expertise was also a limiting factor in cases of PR recurrence where the prognosis was poor, with a median overall survival of 12 months. In this setting, the primary goal of treatment is to maintain or improve QoL without impeding the OS (20, 28).

There is a lack of literature yet proposing a decision algorithm for PIPAC management for patients with OC (29). The additional analyses allowed us to highlight some trends of longer OS and PFS, in favor of the subgroup having performed three or more PIPACs. The multivariate analysis, although on a retrospective cohort, seemed to emphasize, the presence of ascites, the PCI and the number of PIPACs performed as prognostic factors for OS and PFS. As for the number of pipac, we can assume that only patients with a better performance status can complete their three pipac course.

The emergence of PARPi drugs has profoundly changed the prognosis of patients with platinum-sensitive recurrence regardless of their BRCA or HRD mutation status. In our cohort, we did not have the number of platinum-sensitive recurrences or situations where chemotherapy was contraindicated due to toxicity or patient refusal. To date, PIPAC has no place in the treatment armamentarium for PS OC, given the large and effective therapeutic options available for this subgroup. In our cohort, 9–10% of patients with initially unresectable tumors were eligible for CRS. OC with peritoneal involvement remains a complex site to target with less bioavailability to systemic chemotherapy and less distribution throughout peritoneal metastases (23). Vergote et al. showed in their randomized trial that 45% of patients remained unresectable after completing three cycles of carboplatin-paclitaxel as neoadjuvant chemotherapy (30). Combined with systemic chemotherapy, PIPAC could be an option to overcome the risk of peritoneal disease. PIPACOVA is a French phase I dose escalation clinical trial (NCT04811703) with a secondary endpoint of assessing the success rate of conversion to surgery in initially unresectable patients treated with bidirectional chemotherapy if deemed unresectable after three courses. The trial is currently in the recruitment stage. There is currently an Indian phase 3 trial ongoing evaluating the role of PIPAC for recurrent OC PSM, with RECIST morphological assessment as the primary endpoint (31). Interim analysis showed PIPAC with better objective response rates and improved quality of life when compared to chemotherapy arm with acceptable morbidity, which supports our findings (32).

The limitations of our study are its retrospective design, the wide heterogeneity of systemic chemotherapy regimens across centers, and the relatively high rate of missing data for radiological and pathological endpoints. However, it provides a snapshot of the use of PIPAC in patients treated palliatively for ovarian cancer, alone or in combination with systemic chemotherapy.

The administration of PIPAC for patients with PSM from recurrent OC has been confirmed to be safe and associated with low perioperative morbidity and mortality. Future trials will have to determine the place of PIPAC in the therapeutic armamentarium of patients with ovarian cancer and non-met needs, such as unresectable disease, high recurrence number, or platinum-resistant relapse.

## Data availability statement

The raw data supporting the conclusions of this article will be made available by the authors, without undue reservation.

## Ethics statement

The studies involving human participants were reviewed and approved by IRB, Hospices Civils de Lyon, France IRB, CHUV, Lausanne, Switzerland IRB, Zydus Hospital, Ahmedabad, India IRB, Clinica del Pilar, Barcelona, Spain IRB, P.A. Herzen, Thoracoabdominal, Moscow, Russia IRB, University Hospital Tübingen, Tübingen, Germany IRB, University Hospital of Leipzig, Leipzig, Germany IRB, Cancer Institute Montpellier (ICM), Montpellier, France IRB, Ghent University Hospital, Ghent, Belgium IRB, Fondazione Policlinico Universitario A. Gemelli IRCCS, Rome, Italy IRB, Candiolo Cancer Institute, FPO-IRCCS, Candiolo, Italy. The patients/participants provided their written informed consent to participate in this study.

## Author contributions

The authors AK, NB, OG, MH contributed to the conception and design of the study, analysis and interpretation of the data, and drafting and critical revision of the manuscript. AK and NB contributed to data collection and statistical analysis. All authors contributed to the article and approved the submitted version.
