# Supplementary material for: Descriptive review of current practices and prognostic factors in patients with ovarian cancer treated by pressurized intraperitoneal aerosol chemotherapy (PIPAC): a multicentric, retrospective, cohort of 234 patients
Source: Front Oncol. 2023 Aug 24;13:1204886. doi: 10.3389/fonc.2023.1204886 (PMC10484798; doi:10.3389/fonc.2023.1204886)
Supplement: Supplementary file 1 [file DataSheet_1.pdf]

SUPPLEMENTARY MATERIALS

Figure S1: Flow chart of included patients

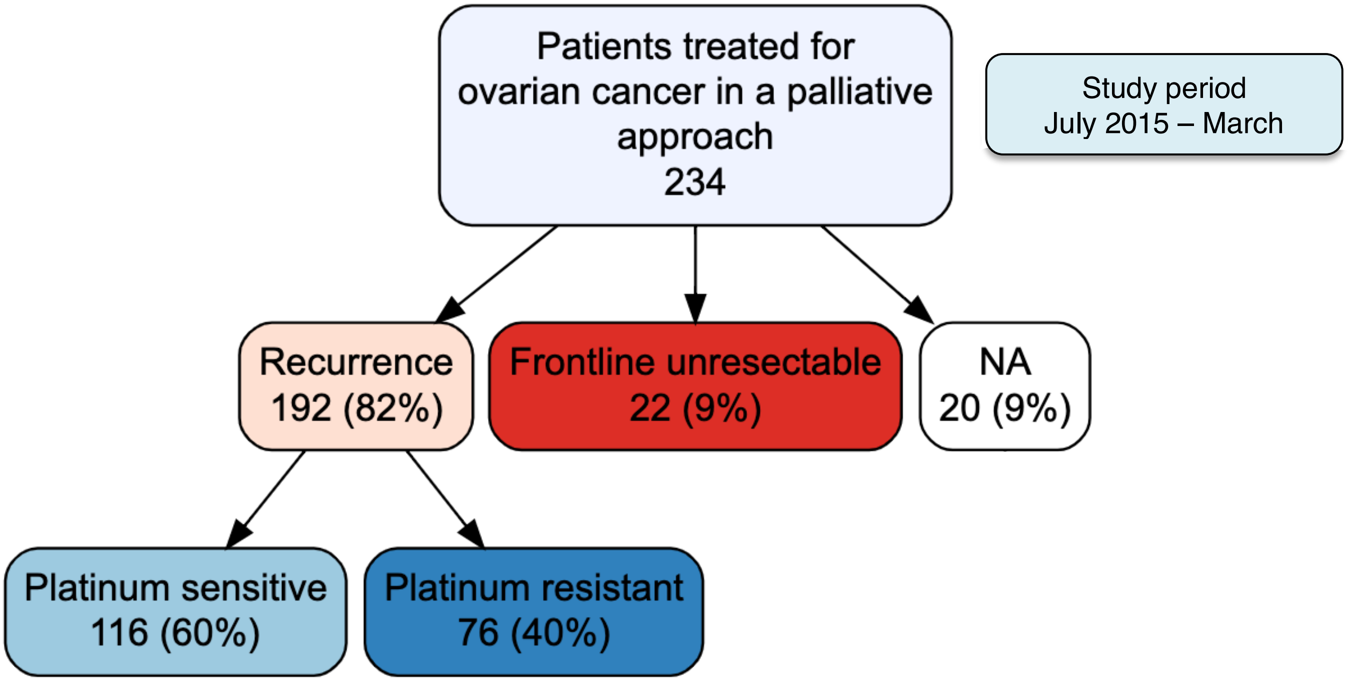

NA: non available data

Figure S2: Overall survival adjusted to number of PIPAC

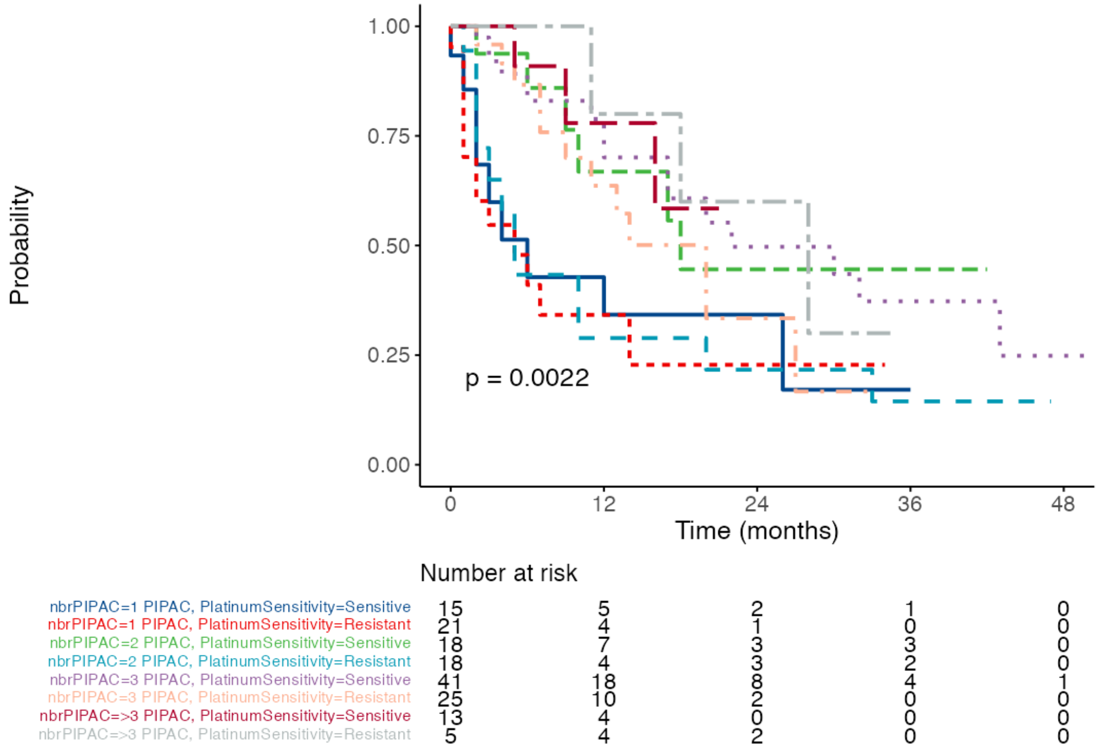

Figure S3: Progression-free survival adjusted to number of PIPAC

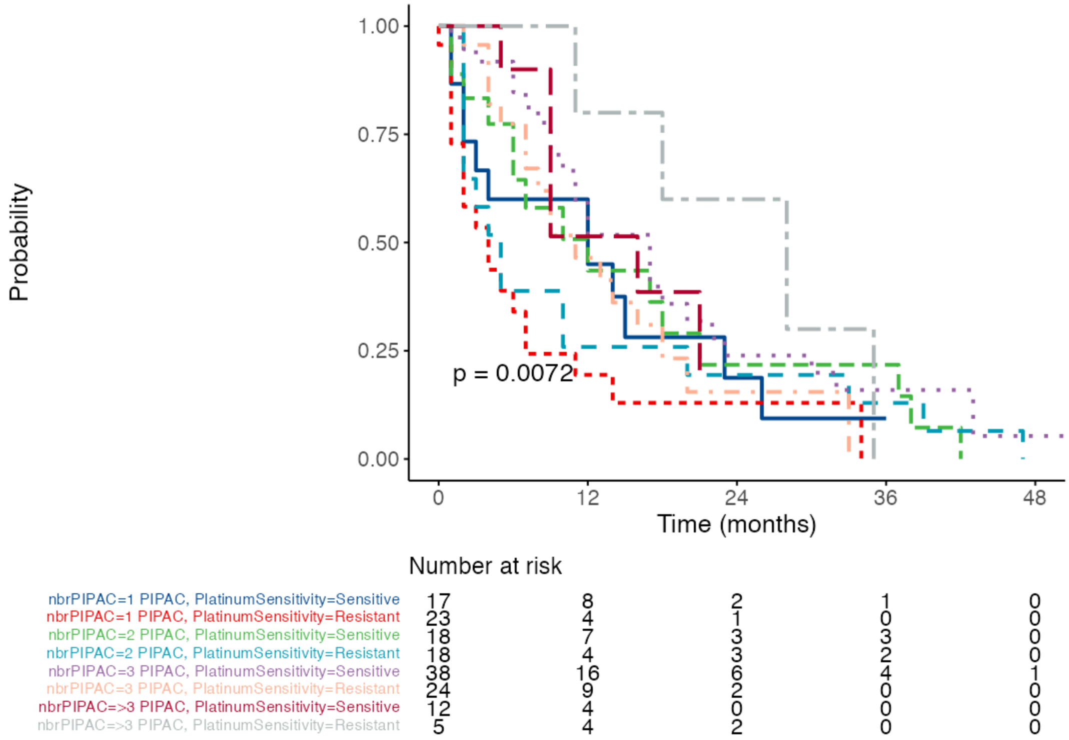

Table S1a: Pre-PIPAC symptoms

Characteristic	N	Platinum sensitive N = 116 (60%)[1]	Platinum resistant N = 76 (40%)[1]	p-value[2]
**Symptoms***	190	49 (42%)	43 (58%)	0.033
Missing		0	2	
**Pain**	177	31 (30%)	26 (36%)	0.36
Missing		11	4	
**Ascitis**	176	22 (22%)	26 (35%)	0.046
Missing		14	2	
**Dysphagia**	172	3 (3.0%)	6 (8.5%)	0.16
Missing		15	5	
**Obstipation**	178	14 (13%)	7 (9.7%)	0.48
Missing		10	4	
**Nausea**	176	17 (16%)	23 (32%)	0.015
Missing		12	4	
**Ascitis, at 1st PIPAC#**	181	0.00 (0.00, 0.20)	0.00 (0.00, 1.10)	0.18
Missing		8	3	

[1] n (%); Median (IQR)
[2] Pearson's Chi-squared test; Fisher's exact test; Wilcoxon rank sum test
* Overall symptoms
# Volume of ascitis in liter

Table S1b: Post-PIPAC symptoms

Characteristic[1]	N	Platinum sensitive N = 116 (60%)[2]	Platinum resistant N = 76 (40%)[2]	p-value[3]
**Symptoms***	128	35 (44%)	21 (44%)	>0.99
Missing		36	28	
**Pain**	122	15 (19%)	13 (30%)	0.19
Missing		38	32	
**Ascitis**	131	17 (20%)	13 (27%)	0.39
Missing		33	28	
**Dysphagia**	119	1 (1.3%)	1 (2.3%)	>0.99
Missing		41	32	
**Obstipation**	123	6 (7.6%)	6 (14%)	0.35
Missing		37	32	
**Nausea**	122	6 (7.7%)	11 (25%)	0.008
Missing		38	32	

[1] Symptoms after 3 (or at least 2) PIPAC cycles
[2] n (%)
[3] Pearson's Chi-squared test; Fisher's exact test
* Overall symptoms

Table S2: Follow-up

Characteristic	N	Platinum sensitive N = 116 (60%)[1]	Platinum resistant N = 76 (40%)[1]	p-value[2]
**Reason to stop PIPAC**	158			0.35
Eligible CRS/HIPEC		8 (8.9%)	7 (10%)	
End of protocole		34 (38%)	16 (24%)	
Poor response		39 (43%)	35 (51%)	
Refusal		3 (3.3%)	5 (7.4%)	
Surgical cause		6 (6.7%)	5 (7.4%)	
Missing		26	8	
**Treatment following PIPAC**	97			0.68
CRS		3 (6.0%)	5 (11%)	
CRS-HIPEC		3 (6.0%)	1 (2.1%)	
Supportive/palliative care		13 (26%)	14 (30%)	
Systemic Chemo		31 (62%)	27 (57%)	
Missing		66	29	
**Progression at follow up**	170	66 (67%)	58 (81%)	0.055
Missing		18	4	

[1] n (%)

[2] Fisher's exact test; Pearson's Chi-squared test

Table S3a: Overall survival predictors with univariate and multivariate Cox regression analysis

Explanatory variables		all, n (%)	HR (univariable)	HR (multivariable)
Platinum sensitivity	Sensitive	41 (46.6)	-	-
	Resistant	47 (53.4)	1.46 (0.77-2.74, p=0.243)	1.39 (0.71-2.74, p=0.336)
Ascitis (before PIPAC)	no	56 (63.6)	-	-
	yes	32 (36.4)	4.32 (2.22-8.40, p<0.001)	4.02 (1.84-8.81, p<0.001)
Cytology (1st PIPAC)	negative	33 (37.5)	-	-
	positive	55 (62.5)	5.26 (2.31-11.97, p<0.001)	3.91 (1.67-9.14, p=0.002)
PCI (1st PIPAC)	≤15	28 (31.8)	-	-
	>15	60 (68.2)	2.21 (1.07-4.56, p=0.032)	2.36 (0.99-5.59, p=0.052)
Obstipation (before PIPAC)	no	80 (90.9)	-	-
	yes	8 (9.1)	3.24 (1.42-7.38, p=0.005)	1.64 (0.66-4.04, p=0.283)
PIPAC (≥ 3 cycles)	no	44 (50.0)	-	-
	yes	44 (50.0)	0.39 (0.21-0.73, p=0.004)	0.30 (0.14-0.63, p=0.002)

Table S3b: Progression-free survival predictors with univariate and multivariate Cox regression analysis

Explanatory variables		all, n (%)	HR (univariable)	HR (multivariable)
Platinum sensitivity	Sensitive	39 (44.3)	-	-
	Resistant	49 (55.7)	1.03 (0.62-1.71, p=0.923)	1.01 (0.58-1.74, p=0.982)
Ascitis (before PIPAC)	no	57 (64.8)	-	-
	yes	31 (35.2)	7.42 (3.79-14.53, p<0.001)	5.22 (2.56-10.62, p<0.001)
Cytology (1st PIPAC)	negative	34 (38.6)	-	-
	positive	54 (61.4)	3.09 (1.71-5.57, p<0.001)	1.96 (1.05-3.67, p=0.035)
PCI (1st PIPAC)	≤15	27 (30.7)	-	-
	>15	61 (69.3)	2.35 (1.29-4.28, p=0.005)	2.50 (1.20-5.20, p=0.014)
Obstipation (before PIPAC)	no	80 (90.9)	-	-
	yes	8 (9.1)	2.44 (1.15-5.18, p=0.021)	1.39 (0.61-3.17, p=0.435)
PIPAC (≥ 3 cycles)	no	46 (52.3)	-	-
	yes	42 (47.7)	0.72 (0.44-1.20, p=0.210)	0.48 (0.27-0.88, p=0.017)
