# Supplementary material for: Descriptive review of current practices and prognostic factors in patients with ovarian cancer treated by pressurized intraperitoneal aerosol chemotherapy (PIPAC): a multicentric, retrospective, cohort of 234 patients
Source: Front Oncol. 2023 Aug 24;13:1204886. doi: 10.3389/fonc.2023.1204886 (PMC10484798; doi:10.3389/fonc.2023.1204886)
Supplement: Supplementary file 2 [file DataSheet_2.docx]

**ISSPP PIPAC study group**

**List of authors :**

- Brigand Cécile MD Strasbourg University Hospital- Hautepierre Hospital Strasbourg University Hospital - Hautepierre Hospital, Department of general and digestive surgery,
- Delhorme Jean-Baptiste MD, PhD Strasbourg University Hospital- Hautepierre Hospital Strasbourg University Hospital - Hautepierre Hospital, Department of general and digestive surgery
- Romain Benoit MD, PhD Strasbourg University Hospital- Hautepierre Hospital Strasbourg University Hospital - Hautepierre Hospital, Department of general and digestive surgery
- Charleux-Muller Diane MD Strasbourg University Hospital- Hautepierre Hospital Strasbourg University Hospital - Hautepierre Hospital, Department of general and digestive surgery
- Bertin Jean-Baptiste MD Strasbourg University Hospital- Hautepierre Hospital Strasbourg University Hospital - Hautepierre Hospital, Department of general and digestive surgery
- Rohr Serge MD Strasbourg University Hospital- Hautepierre Hospital Strasbourg University Hospital - Hautepierre Hospital, Department of general and digestive surgery
- Jäger Tarkan MD, Department of Surgery, Paracelsus Medical University Salzburg, 5020 Salzburg, Austria
- Neureiter Daniel MD, Institute of Pathology, Paracelsus Medical University Salzburg, 5020 Salzburg, Austria
- Schredl Philipp MD ,Department of Surgery, Paracelsus Medical University Salzburg, 5020 Salzburg, Austria
- Weiss Lukas MD "1. Department of Internal Medicine III with Haematology, Medical Oncology, Haemostaseology, Infectiology and Rheumatology, Oncologic Center, Salzburg Cancer Research Institute-Laboratory for Immunological and Molecular Cancer Research (SCRI-LIMCR), Paracelsus Medical University, 5020 Salzburg, Austria 2. Cancer Cluster Salzburg, 5020 Salzburg, Austria"
- Klieser Eckhard MD ,Institute of Pathology, Paracelsus Medical University Salzburg, 5020 Salzburg, Austria
- Emmanuel Klaus MD ,Department of Surgery, Paracelsus Medical University Salzburg, 5020 Salzburg, Austria
- SGARBURA Olivia MD, PhD Institut du Cancer Montpellier 1) Department of Surgical Oncology, Institut du Cancer Montpellier, Montpellier France; 2) IRCM, Institut de Recherche en Cancérologie de Montpellier, INSERM U1194, Université de Montpellier, Institut régional du Cancer de Montpellier, Montpellier, F-34298, France
- BOUILLIN Alix MD Institut du Cancer Montpellier Department of Surgical Oncology, Institut du Cancer Montpellier, Montpellier France
- KHELLAF Lakhdar MD Institut du Cancer Montpellier Department of Pathology, Institut du Cancer Montpellier
- NOUGARET Stéphanie MD, PhD Institut du Cancer Montpellier Department of Radiology, Institut du Cancer Montpellier
- SAMALIN Emmanuelle MD Institut du Cancer Montpellier Department of Medical Oncology, Institut du Cancer Montpellier
- MAZARD Thibault MD, PhD Institut du Cancer Montpellier Department of Medical Oncology, Institut du Cancer Montpellier
- GLEHEN OLIVIER MD, PhD Hôpital Lyon Sud Department of Surgical Oncology, Hôpital Lyon Sud, Hospices Civils de Lyon, Lyon, France; EA 3738 CICLY, Université de Lyon, Lyon, France.
- BAKRIN NAOUAL MD, PhD Hôpital Lyon Sud Department of Surgical Oncology, Hôpital Lyon Sud, Hospices Civils de Lyon, Lyon, France; EA 3738 CICLY, Université de Lyon, Lyon, France.
- KEPENEKIAN VAHAN MD, PhD Hôpital Lyon Sud Department of Surgical Oncology, Hôpital Lyon Sud, Hospices Civils de Lyon, Lyon, France; EA 3738 CICLY, Université de Lyon, Lyon, France.
- ISABELLE BONNEFOY Hôpital Lyon Sud Department of Surgical Oncology, Hôpital Lyon Sud, Hospices Civils de Lyon, Lyon, France; EA 3738 CICLY, Université de Lyon, Lyon, France.
- PARISOT MARLENE Hôpital Lyon Sud Department of Surgical Oncology, Hôpital Lyon Sud, Hospices Civils de Lyon, Lyon, France; EA 3738 CICLY, Université de Lyon, Lyon, France.
- VILLENEUVE LAURENT PhD Hôpital Lyon Sud Service de Recherche et d'Epidémiologie Cliniques , Pôle de Santé Publique, Hospices Civils de Lyon, Lyon, France; EA 3738 CICLY, Université de Lyon, Lyon, France.
- Hübner Martin MD CHUV Lausanne Department of Visceral Surgery, Lausanne University Hospital CHUV, University of Lausanne (UNIL), Lausanne, Switzerland
- Demartines Nicolas MD CHUV Lausanne Department of Visceral Surgery, Lausanne University Hospital CHUV, University of Lausanne (UNIL), Lausanne, Switzerland
- Teixeira-Farinha Hugo MD CHUV Lausanne Department of Visceral Surgery, Lausanne University Hospital CHUV, University of Lausanne (UNIL), Lausanne, Switzerland
- Clerc Daniel MD CHUV Lausanne Department of Visceral Surgery, Lausanne University Hospital CHUV, University of Lausanne (UNIL), Lausanne, Switzerland
- Kefleyesus Amaniel MD CHUV Lausanne Department of Visceral Surgery, Lausanne University Hospital CHUV, University of Lausanne (UNIL), Lausanne, Switzerland
- Dromain Clarisse MD CHUV Lausanne Department of Medical Oncology, Institut du Cancer Montpellier
- Sempoux Christine MD, PhD CHUV Lausanne Department of Medical Oncology, Institut du Cancer Montpellier
- Robella Manuela MD Candiolo Cancer Institute, FPO - IRCCS, Candiolo, Italy Unit of Surgical Oncology, Candiolo Cancer Institute, FPO - IRCCS, Candiolo, Italy.
- Vaira Marco MD Candiolo Cancer Institute, FPO - IRCCS, Candiolo, Italy Unit of Surgical Oncology, Candiolo Cancer Institute, FPO - IRCCS, Candiolo, Italy.
- De Simone Michele MD Candiolo Cancer Institute, FPO - IRCCS, Candiolo, Italy Unit of Surgical Oncology, Candiolo Cancer Institute, FPO - IRCCS, Candiolo, Italy.
- Di Giorgio Andrea MD Fondazione Policlinico Universitario A. Gemelli IRCCS Surgical Unit of Peritoneum and Retroperitoneum, Fondazione Policlinico Universitario A. Gemelli IRCCS, Rome, Italy.
- Ferracci Federica MD Fondazione Policlinico Universitario A. Gemelli IRCCS Surgical Unit of Peritoneum and Retroperitoneum, Fondazione Policlinico Universitario A. Gemelli IRCCS, Rome, Italy.
- Rotolo Stefano MD Fondazione Policlinico Universitario A. Gemelli IRCCS Department of Surgical, Oncological and Oral Sciences, University of Palermo, Palermo, Italy.
- Schena Carlo Alberto MD Fondazione Policlinico Universitario A. Gemelli IRCCS General Surgery, Fondazione Policlinico Universitario A. Gemelli IRCCS, Rome, Italy.
- Inzani Frediano MD Fondazione Policlinico Universitario A. Gemelli IRCCS Surgical Unit of Peritoneum and Retroperitoneum, Fondazione Policlinico Universitario A. Gemelli IRCCS, Rome, Italy.
- Bagalà Cinzia MD Fondazione Policlinico Universitario A. Gemelli IRCCS Division of Medical Oncology, Fondazione Policlinico Universitario A. Gemelli IRCCS, Roma, Italy.
- Babucke Maximilian MD department of general surgery Krankenhaus Barmherzige Brüder Regensburg, Prüfeninger Str. 86, 93049 Regensburg, Germany
- Piso Pompiliu PhD, MD department of general surgery Krankenhaus Barmherzige Brüder Regensburg, Prüfeninger Str. 86, 93049 Regensburg, Germany
- Somashekhar s p MD Manipal Comprehensive Cancer Center, Bengaluru Department of Surgical Oncology
- ASHWIN KR DNB( Surgical Oncology) Manipal Comprehensive Cancer Center, Bengaluru Department of Surgical Oncology
- ROHIT KUMAR DNB( Surgical Oncology) Manipal Comprehensive Cancer Center, Bengaluru Department of Surgical Oncology
- PRIYA KAPOOR MS, DNB (General Surgery) Manipal Comprehensive Cancer Center, Bengaluru Department of Surgical Oncology
- SUSMITA RAKSHIT MBBS,MD ( Pathology) Manipal Comprehensive Cancer Center, Bengaluru Department of Pathology
- AMIT RAUTHAN DM (Medical Oncology) Manipal Comprehensive Cancer Center, Bengaluru Department of Medical Oncology
- BHATT Aditi MS, MCh. Zydus Hospital, Ahmedabad Department of surgical oncology
- SHAIKH Sakina BHMS, MBA Zydus Hospital, Ahmedabad Department of surgical oncology
- PARIKH Loma MD Zydus Hospital, Ahmedabad Department of Pathology
- SHETH Sandeep MD Zydus Hospital, Ahmedabad Department of Pathology
- PANCHAL Amee MD Zydus Hospital, Ahmedabad Department of Radiodiagnosis and Imaging
- Thakkar Shweta MD Zydus Hospital, Ahmedabad Department of Radiodiagnosis and Imaging
- POCARD Marc MD, PhD INSERM, U1275 CAP Paris-Tech 1.    Université de Paris, INSERM, U1275 CAP Paris-Tech, F-75010 Paris, France. 2.    Hepato-Biliary-pancreatic Gastrointestinal Surgery and Liver Transplantation, Pitié Salpêtrière Hospital, AP-HP, F-75013 Paris, France.
- EZZANO Anne-Cécile MD Hôpital Bégin Department of visceral surgery, BEGIN Military Hospital, 69, avenue de Paris, 94160 St Mandé, France
- AIME Adeline MD Hôpital Bégin Department of visceral surgery, BEGIN Military Hospital, 69, avenue de Paris, 94160 St Mandé, France
- EVENO Clarisse MD, PhD CHU Lille Department of Digestive and Oncological Surgery Claude Huriez University Hospital, 59000 Lille, France; UMR-S1277-CANTHER laboratory "Cancer Heterogeneity Plasticity and Resistance to Therapies", Lille, France.
- NOIRET Barbara MD CHU Lille Department of Digestive and Oncological Surgery Claude Huriez University Hospital, 59000 Lille, France
- Khomiakov Vladimir PhD P.A. Hertsen Moscow Research Oncological Institute - Branch of the National Medical Research Centre of Radiology., Moscow, Russia P.A. Hertsen Moscow Research Oncological Institute - Branch of the National Medical Research Centre of Radiology., Moscow, Russia
- Ryabov Andrey PhD P.A. Hertsen Moscow Research Oncological Institute - Branch of the National Medical Research Centre of Radiology., Moscow, Russia P.A. Hertsen Moscow Research Oncological Institute - Branch of the National Medical Research Centre of Radiology., Moscow, Russia
- Utkina Anna PhD P.A. Hertsen Moscow Research Oncological Institute - Branch of the National Medical Research Centre of Radiology., Moscow, Russia P.A. Hertsen Moscow Research Oncological Institute - Branch of the National Medical Research Centre of Radiology., Moscow, Russia
- Aksenov Sergey MD P.A. Hertsen Moscow Research Oncological Institute - Branch of the National Medical Research Centre of Radiology., Moscow, Russia P.A. Hertsen Moscow Research Oncological Institute - Branch of the National Medical Research Centre of Radiology., Moscow, Russia
- Bolotina Larisa PhD P.A. Hertsen Moscow Research Oncological Institute - Branch of the National Medical Research Centre of Radiology., Moscow, Russia P.A. Hertsen Moscow Research Oncological Institute - Branch of the National Medical Research Centre of Radiology., Moscow, Russia
- Kaprin Andrey PhD P.A. Hertsen Moscow Research Oncological Institute - Branch of the National Medical Research Centre of Radiology., Moscow, Russia P.A. Hertsen Moscow Research Oncological Institute - Branch of the National Medical Research Centre of Radiology., Moscow, Russia
- Willaert Wouter MD, PhD Ghent University department of human structure and repair, Laboratory of Experimental Surgery
- Cosyns Sarah PhD, MSc Ghent University department of human structure and repair, Laboratory of Experimental Surgery
- Akhayad Soumaya BSN Ghent University Hospital department of gastro-intestinal surgery
- Ceelen Wim MD, PhD Ghent University department of human structure and repair, Laboratory of Experimental Surgery
- Gockel Ines MD University Hospital Leipzig "Department of Visceral, Transplant, Thoracic and Vascular Surgery"
- Jansen-Winkeln Boris MD University Hospital Leipzig "Department of Visceral, Transplant, Thoracic and Vascular Surgery and Department of General, Visceral and Oncological Surgery, St. Georg Hospital, Leipzig, Germany"
- Thieme René PhD University Hospital Leipzig "Department of Visceral, Transplant, Thoracic and Vascular Surgery"
- Schierle Katrin MD University Hospital Leipzig Institute of Pathology
- Moulla Yusef MD University Hospital Leipzig Department of Visceral, Transplant, Thoracic and Vascular Surgery
- Mehdorn Matthias MD University Hospital Leipzig Department of Visceral, Transplant, Thoracic and Vascular Surgery
- Abba Julio MD Centre Hospitalier Universitaire Grenoble Alpes Service de chirurgie Digestive
- Trilling Bertrand MD, PhD Centre Hospitalier Universitaire Grenoble Alpes Service de chirurgie Digestive
- Tidadini Fatah msc Centre Hospitalier Universitaire Grenoble Alpes Service de chirurgie Digestive
- Bonne Aline MD Centre Hospitalier Universitaire Grenoble Alpes Service de chirurgie Digestive
- Arvieux Catherine MD, PhD Centre Hospitalier Universitaire Grenoble Alpes Service de chirurgie Digestive
- Orry David MD Centre Georges François Leclerc
- Basso Valeria MD Centre Georges François Leclerc
- Ghiringhelli François MD, PhD Centre Georges François Leclerc
- ESCAYOLA Cecilia MD HOSPITAL EL PILAR QUIRON SALUD Quenet-Torrent Institute
- TORRENT Juan José MD HOSPITAL EL PILAR QUIRON SALUD Quenet-Torrent Institute
- ALYAMI Mohammad MD Department of General Surgery and Surgical Oncology , King Khalid Hospital , Najran , Saudi Arabia
- Alqannas Mashhour MD Department of General Surgery and Surgical Oncology , King Khalid Hospital , Najran , Saudi Arabia
- CORTÉS GUIRAL Delia MD, PhD Department of General Surgery and Surgical Oncology , King Khalid Hospital , Najran , Saudi Arabia
- Alammari samer MD Department of General Surgery and Surgical Oncology , King Khalid Hospital , Najran , Saudi Arabia
- Bashanfer Galal MD Department of Pathology King Khalid Hospital , Najran , Saudi Arabia
- Alshukami Anwar MD Department of Radiology King Khalid Hospital , Najran , Saudi Arabia
- Reymond Marc A. MD Department of General and Transplant Surgery, University of Tuebingen
- Solass Wiebke MD Department of Pathology, University Hospital Tuebingen
- Nadiradze Giorgi MD Department of General and Transplant Surgery, University of Tuebingen
